# Let us tell you how to train us! experiences of children and youth with the Wheelchair Skills Training Program and their preferences for training delivery: a descriptive qualitative study

**DOI:** 10.3389/fresc.2026.1924405

**Published:** 2026-09-03

**Authors:** Béatrice Ouellet, Sofia So, Tatiana Dib, Paula W. Rushton, Margaux Hebinck, Sarah A. Moore, Kelly P. Arbour-Nicitopoulos, R. L. Kirby, François Routhier, Krista L. Best

**Affiliations:** 1 Faculty of Medicine, School of Rehabilitation Sciences, Université Laval, Quebec City, QC, Canada; 2 Center for Interdisciplinary Research in Rehabilitation and Social Integration, Santé Québec Capitale-Nationale – Universitaire, Quebec City, QC, Canada; 3 Faculty of Health, School of Health and Human Performance, Dalhousie University, Halifax, NS, Canada; 4 School of Rehabilitation, Université de Montréal, Montréal, QC, Canada; 5 CHU Sainte-Justine Research Centre, Montréal, QC, Canada; 6 Faculty of Health, School of Occupational Therapy, Dalhousie University, Halifax, NS, Canada; 7 Faculty of Medicine, Department of Pediatrics, Dalhousie University, Halifax, NS, Canada; 8 Pediatric Rehabilitation, IWK Health, Halifax, NS, Canada; 9 Faculty of Kinesiology and Physical Education, University of Toronto, Toronto, ON, Canada; 10 Division of Physical Medicine and Rehabilitation, Dalhousie University, Halifax, NS, Canada

**Keywords:** assistive technology (AT), lived experience, pediatrics, qualitative study, rehabilitation, training, wheelchairs

## Abstract

**Introduction:**

The Wheelchair Skills Training Program (WSTP) has demonstrated statistical and clinical effectiveness in improving manual wheelchair skills among pediatric manual wheelchair users (PMWUs). However, uptake of this program remains limited in pediatric rehabilitation settings, partly due to clinicians’ uncertainties about its suitability for playful and developmentally appropriate training. Despite children's and youths’ right to engage in discussions regarding their rehabilitation, PMWUs’ lived experiences receiving the WSTP have not yet been explored. The aim of this study was to explore PMWUs’ experiences with and preferences regarding the WSTP to inform the delivery of this program in pediatric rehabilitation.

**Methods:**

A descriptive qualitative study was conducted with a maximum variation sample of PMWUs recruited from a randomized controlled trial evaluating the WSTP in pediatrics. Online or in-person semi-structured interviews, informed by the Template for Intervention Description and Replication checklist for rehabilitation intervention reporting, explored experiences with and preferences regarding WSTP delivery. Interview procedures were individualized using a toolkit of strategies to support expression and engagement. The framework method guided deductive thematic analysis.

**Results:**

Three overarching themes emerged from the 32 PMWUs’: 1) *It*'s *true that not many kids know how to use their wheelchair properly, so I think training is really a good thing*, highlighted the perceived value of the WSTP for improving mobility, confidence, risk awareness, autonomy, and participation; 2) *The trainer lets you practice everything that you want*, reflected how trainers can facilitate mobility progress and engagement through collaborative goal-setting, the use of learning strategies, and the integration of skill practice into enjoyable and appropriately challenging activities; and 3) *It depends on the person. Every child, every person with a disability or mobility aid is different. We're not all the same*, emphasized the need to tailor training sessions to individual needs and preferences.

**Conclusion:**

PMWUs’ positive experiences with the WSTP revealed that the program aligns with their training preferences and supports mobility progress, suggesting its suitability in pediatric rehabilitation settings.

## 1 Introduction

The World Health Organization wheelchair provision guidelines (2023) outline a four-step process (select, fit, train, and follow-up) (1). Training involves teaching individuals to safely and effectively perform manual wheelchair (MWC) skills to achieve self-directed mobility (i.e., mobility in the manner and at the time of their choice) (1, 2). Article 20 of the Convention on the Rights of Persons with Disabilities identifies training as a means of supporting the right to self-directed MWC mobility (3). Among children and youth who use MWC [pediatric manual wheelchair users (PMWUs)], training may also positively influence participation in activities that contribute to reaching developmental milestones, health, and quality of life (1, 4–6).

Despite its acknowledged importance, MWC skills training is not part of usual care in pediatric rehabilitation settings. In fact, 37% of clinicians do not offer MWC skills training to PMWUs because they report lacking resources to guide intervention delivery (7–9). The Wheelchair Skills Training Program (WSTP) is a resource suggesting skill-specific techniques and a set of ten strategies (i.e., Trainer's 10 tools) to train 30 indoor, community and advanced MWC skills based on wheelchair users’ characteristics, the skill being practiced, the wheelchair, and the setting (10). Although evidence supports the clinical and statistical effectiveness of the WSTP in improving MWC skills capacity (6), as well as its positive influence on MWC use self-efficacy and satisfaction with participation in MWC-related activities (5, 6, 11–15), its uptake in pediatric rehabilitation settings remains limited (8). Occupational therapists’ initial impressions of the WSTP suggested that it was insufficiently tailored to support playful and developmentally appropriate training for PMWUs (8). Concerns were raised about whether practicing certain skills would be engaging, appropriately challenging, and safe for this population (8).

However, no studies have yet documented PMWUs’ experiences of or preferences in training with the WSTP. As recipients of training, their input is essential to understanding whether the program meets their needs and which components, if any, require improvement. Children and youth with disabilities, including PMWUs, are rarely involved in discussions about their rehabilitation and healthcare services or asked to share their experiences and preferences regarding intervention delivery (16–19). In fact, researchers often rely on proxies (e.g., caregivers and clinicians) because of the perceived difficulty of eliciting the perspectives of children and youth with disabilities directly, despite evidence that their views may differ (16–18). Including the voices of PMWUs in discussions about MWC skills training may promote engagement in training and adherence, thereby supporting positive mobility and participation outcomes. It also supports the rights of children and youth to be heard and participate in decisions affecting their lives (3, 20), and aligns with the disability rights principle “Nothing About Us Without Us” (21).

In this context, this study aimed to explore PMWUs’ experiences with and preferences regarding the WSTP to inform the delivery of this program in pediatric rehabilitation settings.

## 2 Methods

### 2.1 Research paradigm, design, and framework

This study was grounded in a constructivist paradigm which recognizes the multiplicity of perspectives and conceptualizes experience as lived, socially constructed, and context-dependent, with knowledge co-constructed through interactions between PMWUs and researchers (22, 23). A descriptive qualitative design was used, which allows researchers to answer questions about the who, what, and where of events or experiences (24). It is thus well suited to exploring how individuals perceive a service, why they use it, and the factors that facilitate or hinder their experiences of care (25, 26). Consistent with this paradigm and design, this study explored diverse PMWU perspectives through flexible semi-structured interview methods that facilitated sharing lived experiences related to specific components of WSTP delivery and the perceived benefits they have on self-directed mobility and participation. The Template for Intervention Description and Replication for rehabilitation intervention reporting (TIDieR-Rehab) checklist, which includes 15 items for describing components of a rehabilitation intervention to facilitate its replication, served as framework for data collection and analysis (27). The COnsolidated criteria for Reporting Qualitative (COREQ) research checklist was followed (28).

### 2.2 Researchers’ positionality

The research team included graduate students and researchers in the field of pediatric rehabilitation, wheelchair mobility, adapted physical activity, and implementation science. BO has prior clinical experience (<1 year) as a pediatric occupational therapist in the speech delay program at CIUSSS-CN working with children with communication difficulties. TD has more than 15 years of experience as a pediatric occupational therapist in seating, mobility, and assistive technology at the Centre de readaptation Marie-Enfant (CRME) and affiliated schools. BO, SS, and SM delivered adapted physical activity programs to PMWUs. In addition, BO, SS, TD, and MH have delivered WSTP-based interventions to PMWUs, including the intervention documented in this study (6, 29). Through these clinical, community, and training experiences, BO, SS, MH, TD and SM had prior relationships with PMWUs, which may have influenced data analysis and interpretation. Some members have also contributed to the development and evolution of the WSTP. RLK, PR, FR, and KB have over 25 years of research experience focused on improving wheeled mobility through WSTP training. They are members of the Wheelchair Skills Program Editorial Board, with RLK as Chair, PR serving as Deputy Chair (Academic), and FR as Chair of the French-Canadian subcommittee. PR also chairs the WSP-Pediatric Subcommittee, an international clinician–researcher team working to enhance the pediatric content of the WSTP protocol. BO, TD, SM, and KB are also members of this subcommittee. Such involvement shaped the team's perspective on the importance and benefits of evidence-based wheelchair skills training, influencing the questions asked and the lenses with which data were analysed and interpreted (23). Recognizing potential bias stemming from these relationships and involvements, team members engaged in ongoing reflexivity throughout data collection and analysis (23), regularly asking questions such as, “Would someone unfamiliar with the WSTP or this PMWU interpret the data in the same way?”. Concurrently, the entire team was strongly committed to empowering PMWUs to voice their lived experiences and authentically amplify their perspectives, thereby informing future training interventions. This commitment helped balance potential biases, ensuring that PMWUs’ voices rather than researcher assumptions guided data collection, analysis, and interpretation.

### 2.3 Ethical considerations

Ethical approval was obtained from the Research Ethics Boards at each study site (Quebec City and Montreal: #MP-13-2022-2479; Halifax: #1028090). Prior to the interviews, written informed consent or assent (verbally or through non-verbal communication methods) was obtained from all participants. Assent was also continuously verified during interviews (e.g., persistent engagement), with the option to stop at any time without consequences.

### 2.4 Participants

To capture a diversity of experiences with the WSTP, a maximum variation sample of PMWUs was recruited from a multi-site, pragmatic, wait-list randomized controlled trial (RCT) evaluating the effectiveness of the WSTP. All PMWUs enrolled in the RCT who had completed the intervention or withdrawn from it were eligible to participate. The eligibility criteria were to have participated in at least one WSTP training session, either as part of the experimental or wait-list control group, and to be able to communicate in French or English, either verbally or through a non-verbal mode of communication (e.g., augmentative and alternative communication). Among eligible PMWUs, participants were purposefully selected by people who provided training to ensure variation across: demographic characteristics (e.g., diagnoses, gender, age); manual wheelchair use [e.g., MWC experience, frequency of use (sporadic, occasional, daily)]; and intervention experiences [e.g., site (Quebec, Montreal and Halifax), caregiver attendance or not, training settings (rehabilitation and real-life settings)]. Selected PMWUs or their caregivers were contacted by phone to invite them to participate in the interview a few days after completing (or declining to complete) the RCT assessment conducted following their participation in WSTP training, to support accurate recall of their experience.

### 2.5 Description of the intervention

The intervention, described in detail in Best et al. (2023), consisted of up to 12 individual training sessions (45–60 min each) with the WSTP, delivered at a target frequency of one to two times per week (29). PMWUs were the target recipients of the intervention, but caregivers who wish to accompany them were permitted to observe training sessions. Trainers were graduate students, pediatric occupational, physical or recreation therapists, and an undergraduate student in the field of social work, all of whom received WSTP training. Skills trained were based on wheelchair-related participation goals established with PMWUs and/or their caregivers using the Wheelchair Outcome Measure for Young People (30). Training sessions followed a general structure, including a discussion of mobility progress and goals, a warm-up, skill practice, and a cool-down, that was personalized to each PMWU. Training sessions could be conducted in various settings (i.e., rehabilitation centre or research center, home, daycare, school, or community settings such as malls), as selected by PMWUs and/or their caregivers based on participation goals and family logistics.

### 2.6 Data collection

Sociodemographic and manual wheelchair use information were obtained from the baseline sociodemographic questionnaire of the RCT (6, 29). Descriptive information about PMWUs’ training experiences (e.g., number of training sessions, training settings, caregiver attendance) were obtained from the training logs completed by RCT trainers after each training session. Thirty- to 60-min semi-structured interviews were conducted with PMWUs either online or in-person, according to their preferences, at the Quebec City and Halifax sites, and exclusively online at the Montreal site due to resource constraints. The interview guide (see Supplementary File 1) included 22 questions and probes addressing the why, who, when, what, who provided, how, where, how much, personalisation, and harm items of the TIDieR-rehab checklist to explore PMWUs’ experiences in the WSTP and their training preferences (27). Over the course of the study, the interview guide was iteratively refined to clarify questions that were not well understood or that PMWUs had difficulty answering. Questions and probes were also added as training preferences emerged (e.g., “What do you think about being trained by a skilled wheelchair user?”) to explore topics not spontaneously raised by PMWUs. Inspired by Teachman and Gibson's methodological approach to supporting the expression and engagement of children and youth with disabilities in interviews, the data collection process was individualized for each PMWU through collaboration with caregivers and/or trainers and the use of a toolkit of customizable interview strategies (31).

#### 2.6.1 Collaborating with caregivers and trainers

Interviewers contacted caregivers and/or trainers to identify strategies facilitating communication with PMWUs. Discussions with trainers also helped verify the interpretation of training log data and obtain additional information about training experiences to contextualize interview questions. Caregivers could support their child's interview by rephrasing questions using familiar words, providing context, and clarifying answers, such as those expressed through gestures or vocalizations. Their role was explained before the interview and reiterated as needed to ensure they supported their child's expression without speaking on their behalf. Caregivers had the opportunity to share their perceptions as part of another study.

#### 2.6.2 Toolkit of customizable interview strategies

The standard question–answer interview format was used in combination with strategies designed to foster expression (see Table 1). These were selected prior to the interview in collaboration with caregivers and/or trainers and PMWUs themselves (if they had the cognitive ability to understand them). During the interview, the interviewer could draw on strategies from the toolkit as needed or stop using any of them, adapting to the course of the discussion.

**Table 1:** Toolkit of strategies supporting PMWUs’ expression and engagement in the interview.

Strategies	Description of the “how” and “why” strategies were used in the interviews
Talking cards game (31)	*How:* Six cards are turned over, and PMWUs select them, one-by-one, to reveal the hidden image (e.g., animals, sports, music). Taking turns, PMWUs and interviewers asked themselves questions about the image. *Why:* Icebreaker activity to build rapport; Tailor the interview to PMWUs’ interests (e.g., listening to a favorite song to take a break); Reduce power differentials by fostering a sense of control and comfort (e.g., encouraging questions and choices). *Only used when developmentally appropriate; not used in Halifax, as the interviewer knew PMWUs.
Drawing-based interview (45)	*How:* PMWUs were provided with arts and crafts materials (coloured paper, pencils, scissors, glue, and stickers) and asked to depict what they had in mind when thinking about manual wheelchair skills training. Probes included asking them to draw elements they liked most or least about training. They had up to 20 min to complete the drawing and could ask the interviewer for assistance if needed. The focus was not on artistic quality, but on PMWUs’ explanations of the depicted elements. Questions could be asked by the interviewer throughout the drawing process and afterward. The drawing could be modified during the discussion as reflections on the experience evolved. If the interview was conducted online, PMWUs were asked to complete their drawing beforehand. *Why:* Provide time and a means to reflect about their training experience and organize their ideas visually; Support expression of the elements that matter most in the training experience; Enhance engagement and motivation by using an enjoyable, activity-based method.
Vignettes-based interview (31)	*How:* PMWUs were presented with a hypothetical peer of similar age, interests, and mobility goals who was about to receive the intervention in the coming weeks. Questions focused on the perceived benefits of training for this peer and on which components of the intervention should be maintained or modified to support an enjoyable training experience and enhance mobility progress. *Why:* Mitigate social desirability bias by encouraging PMWUs to focus on improving a peer's experience rather than evaluating their own. This perspective was expected to facilitate more open and honest feedback about aspects of training they disliked or perceived could be improved.
Go along interview (46)	*How:* PMWUs were invited to guide the interviewer through the training setting, showing activities they did, skills they practiced, obstacles they navigated, and materials used, as if in a training session. Questions were asked along the way or when stopping at places identified by PMWUs. *Why:* Explore training preferences using both demonstration and verbal explanation; Provide a concrete understanding of the training context and activities; Support recall of experiences and associated emotions; Enhance engagement by allowing PMWUs to show what they did. *Only used for in-person interviews
Option choice questions	*How:* Questions were reworded to provide two to three response options (e.g., Did you prefer training at the rehabilitation centre or home?) *Why:* To facilitate expression among PMWUs with limited speech abilities or who use augmentative and alternative communication.

### 2.7 Data analyses

Sociodemographic, manual wheelchair use, and training information for each participant were summarized descriptively. A deductive thematic approach guided by the Framework Method was used to analyse the interviews (32). This method allowed coding and charting of the data into the categories of a matrix, providing a structured and visual summary of the dataset (32). Interview recordings were listened to, and responses to questions were transcribed verbatim. Drawings were not analyzed, as the focus was on PMWUs’ explanations of the visual illustrations they produced. For the go-along strategy, transcripts were supplemented with observational notes when PMWUs showed or demonstrated elements rather than verbally described them. All transcripts and observational notes were verified against the recordings by an undergraduate student in occupational therapy and a graduate student (BO or SS). The TIDieR-Rehab checklist was used as the analytical framework, with the why, who, when, what, who provided, how, where, how much, how challenging, progression, personalisation, and harms items serving as analytical categories. Within each category, codes were developed to capture specific elements of the intervention description (e.g., What: core components of the WSTP, such as tools supporting skill learning, including practice, instruction, and demonstration) and delivery (e.g., How: individual or group format; Where: rehabilitation or real-world settings). BO conducted initial coding of the first ten interviews to refine the analytical framework, classifying data segments within the predefined TIDieR-Rehab categories and associated codes (27) in NVivo (version 15.5) (33). Questions and uncertainties were discussed with MH and SS, with remaining concerns addressed through discussion with research supervisors (KB and PR), thus enhancing confirmability (34). The refined analytical framework was then applied to all interviews by BO. Throughout the analysis, relevant data segments were charted into a matrix organized by TIDieR-Rehab categories and corresponding codes (27, 32). Then, categories sharing related content were grouped into themes, named using PMWUs’ quotations capturing the overarching message in participants’ own words. Eight team meetings were held to reflect on the matrix, resolve coding and charting issues, collaboratively interpret the data, and discuss reporting of the results, thereby enhancing the credibility and confirmability of the findings (34). Data were analyzed in their original language. Quotes from French-speaking PMWUs presented in the results were translated into English by bilingual members of the research team (BO, TD, KB).

## 3 Results

### 3.1 Participants

Thirty-two PMWUs participated in the interviews, of whom 30 were fully analysed and one partially due to concerns regarding the credibility of two participants’ responses (e.g., contradictory answers, repetition of interviewers’ questions, and verbatim echoing of caregivers’ wording), limiting interpretation. Sociodemographic, manual wheelchair use, and intervention data of the participants are presented in Table 2. All PMWUs attended the 12 WSTP sessions, except one who stopped the intervention after completing 9 sessions due to scheduling and health-related reasons. Ten interviews were conducted online and 22 in-person. Two twin PMWUs (QY8a and QY8b), who received the intervention jointly at their family's request, participated in a joint interview, both responding to each interview question. Among the toolkit strategies, the talking cards icebreaker activity, the vignette, the drawing, and the go-along methods were used with five, six, one, and two PMWUs, respectively. Only two interviews were entirely structured with a vignette and one with the go along method, with most strategies being used as needed to elicit PMWUs’ responses or support their engagement. The participants’ quotes included in the text below are identified by site (Montreal = M; Quebec City = Q; Halifax = H), age group (children = C; youth = Y), and participant number (e.g., QY0: Quebec, youth, pilot; MK7: Montreal, child, participant 7).

**Table 2:** Sociodemographic, manual wheelchair use, and intervention data.

Categories	Frequency (%)
Gender	
Girls	12 (37.5)
Boys	20 (62.5)
Age (in years)	
*Mean (SD)* *=* *12.3 (4.5)*	
3–5	2 (6.2)
6–11	14 (43.8)
12–18	15 (46.9)
19–21	1 (3.1)
Diagnosis	
Cerebral palsy and encephalopathy	11 (34.4)
Spina bifida	2 (6.2)
Neuromuscular diseases (e.g., spinal muscular atrophy)	7 (21.9)
Musculoskeletal conditions (e.g., arthrogryposis, amputation)	3 (9.3)
Other conditions (e.g., neurological, syndromes)	9 (28.2)
Experience using a MWC (in years)	
*Mean (SD)* *=* *7.1 (4.1)*	
0–1	2 (6.2)
2–5	11 (34.4)
≥6	19 (59.4)
Daily MWC use	
0 (i.e., sporadic and occasional users)	2 (6.2)
1–5	9 (28.2)
≥6	21 (65.6)
Previously received MWC skills training	
Yes	7 (21.9)
No	25 (78.1)
Site at which they received training	
Quebec City	12 (37.5)
Montreal	5 (15.6)
Halifax	15 (46.9)
Caregiver attendance (number of sessions)	
*Mean (SD)* *=* *7.8 (4.7)*	
0	3 (9.3)
1–11	15 (46.9)
12	14 (43.8)
Training settings (number of PMWUs)	
Trained at rehabilitation or research centre only	8 (25)
Trained in real-word settings only	10 (31.3)
Trained in rehabilitation/research centre and real-world	14 (43.8)

### 3.2 Theme 1: it's true that not many kids know how to use their wheelchair properly, so I think training is really a good thing [MC7]

This theme highlights the rationale (why) for delivering the WSTP from the perspective of PMWUs, who reported benefits in mobility, confidence, risk awareness, autonomy, and participation as a result of their training experience. All participants expressed interest in recommending the program to others.

#### 3.2.1 Why? progressing in mobility while still needing practice

PMWUs perceived that the WSTP enabled them to learn skills they had not thought possible: “*Go down an incline in a wheelie: I thought it was impossible to do, physically speaking, with gravity pulling you backward [MY7]*”. Practicing skills they were unable to perform and improving existing ones using safer, and less energy-demanding techniques were also reported. For example, when picking up objects from the floor, MY3 explained trying to remember to position his casters to prevent falls and leaning on one side rather than getting out of his wheelchair. While some skills were perceived as mastered—“*easy peasy lemon squeezy, opening doors [HC2]*”-, PMWUs needed further practice for skills that remained difficult, and noted that established habits were hard to change. In this regard, a 20-year-old PMWU who had used a MWC since the age of four explained: “*I try to pay attention to what we practiced. I try to propel correctly, but I often go back to my old wheeling style. I still need [trainer's name] to tell me to do this and that [QY7]*”. As such, except QY0, who was satisfied with his progress, all PMWUs expressed the desire to receive follow-up training: “*I could learn more things. For example, in the training, I wasn't able to climb the 10-centimeter curb, but with another, I'll try again, and eventually, I'll be able to do it [MC1]*”. Moreover, a few PMWUs who used power-assist add-ons found benefits from practicing skills both with and without them, noting slight variations in skill execution (e.g., ascending curbs with enhanced speed). Similarly, those using two wheelchairs described improvements training with both, such as QC5 explaining improving the wheelie skill that she had more difficulty performing with her folding MWC than with her all-terrain MWC with a free wheel: “*Before, I was not able to do a wheelie with the wheelchair I use at school [folding frame]. Now, I can roll forward, backward. I just had to tip a little more backward [with the folding frame], but I was always doing it with my other chair [all terrain MWC with a free wheel]. I did not know I had to be more backward*”*.*

#### 3.2.2 Why? growing pride, confidence and risk awareness

PMWUs were “*really proud [QC1]*” to witness their progress throughout the sessions with some feeling like they became experts of wheelchair skills, such as “*the expert of the incline [QY2]*”. Practicing with a trainer who demonstrated skills and ensured safety, helped PMWUs feel “*way less terrified [HC1]*” and more confident and capable of performing skills: “*Well, I feel more confident in general when it comes to going up curbs or navigating bumpy grounds. I know I can do it. It would take me some time, but I could do it. By learning skills, you know you can do it [QC4]*”. Moreover, an 18-year-old PMWU with 14 years of experience using a manual wheelchair reflected on balancing risks while considering his confidence when thinking about descending stairs independently after practicing this skill: “*I’m aware of the risk of falling, but I’m more confident than just worrying about the danger. I’ve done this before [in training sessions], I’ve done it a lot [in training sessions], so it's pretty easy. I also tell myself: Now I’m all alone. It's not the same. If I fall, there might be someone there, but not immediately. I trust my own confidence more than I fear the danger. [MY7]*”.

#### 3.2.3 Why? training mobility skills fosters autonomy and participation

Many PMWUs perceived that the skills they learned and the confidence they gained improved their autonomy and participation at home, school, work, and in the community, as illustrated by QY0: “*At school, I can now take the elevator on my own with my power assist. [Trainer's name] taught me, I can transfer from the bed to the wheelchair by myself, without help. […] During the 25-min recess, I can go around the second floor at school.*” However, a few PMWUs reported difficulties applying skills learned from one setting to another, which limited their use in day-to-day life: “*I think that the day I practice in my neighborhood, I’ll have to start again a bit, because it won’t be the same. My brain has trouble switching places [QY7]*”. When asked about changes in his participation, MY3 mentioned having limited opportunities to leave his house but emphasized that the WSTP enabled him to do so during the training sessions: “*To be honest, I don’t go out very much… Um, this [the WSTP] kind of got me out of the house a bit, because I spend a lot of time at home. Once in a while, I go out, but like, not every day… I enjoyed exploring the neighborhood a little*”. Sporadic and occasional PMWUs reported not yet applying new skills in day-to-day life but feeling more prepared to do so when they would need them. Table 3 provides examples of activities on which PMWUs reported a positive impact of wheelchair skills training.

**Table 3:** Examples of activities reported to increase autonomy or participation.

Life settings	Examples of activities reported to increase autonomy or participation
Home	- Transfer from wheelchair to the bed and sofa (and vice versa)
Neighbourhood	- Overcome speed bumps and propel on slanted roads to navigate streets;@-Roll on the cycling lane with the power-assist add-ons;@-Self-propel on longer distances (higher endurance) and more rapidly (e.g., from home to school).
School and daycare	- Navigate inside the daycare (e.g., roll in the hallway, open doors) and school (e.g., take the elevator alone and descend stairs when it is broken); - Navigate in the schoolyard (e.g., wood chips in the playground) - Physical education: • Do wheelies to play jump rope; • Remove wheelchair components (e.g., headrest) to make it lighter and facilitate participation in physical activities; - Play with friends (e.g., wheelie competitions); - Skills and confidence in overcoming obstacles (e.g., ascending inclines) facilitate PMWUs’ transition to new school environments (e.g., high school, post-secondary education).
Community	- Overcome obstacles (e.g., curbs, gravel, inclines) without help when hanging out with friends, going to the restaurant and mall, and during school outdoor outings; - Self-propel from the parking lot to healthcare settings for medical appointments.
Work	- Get over the curb at the entrance of the grocery store where they work.

Despite increased capacity to move on their own, most PMWUs described still needing supervision or assistance, especially for energy conservation, weather conditions (e.g., cold and snow), crowded settings, and safe navigation of challenging obstacles. Moreover, PMWUs explained that caregivers and educators influence the opportunities they have to use the wheelchair skills learned during the WSTP, either supporting or restricting autonomy. For example, QY3, who received assistance with indoor and outdoor mobility before participating in the WSTP, said: “*Mommy didn’t want to push me when I wanted her to because it was hard, but she said try to work it out by yourself*”, whereas QY5 and QY8a respectively stated “*My mom does not like to wait, so she pushes me*” and “*At school, they are protective so we can’t practice. People fear that we will hurt ourselves*”*.* In contrast, two adolescents who made substantial improvements in mobility post-WSTP reported increased independence and reduced reliance on caregivers: “*It's really nice to know that you can be independent and don’t need help. It makes things easier for yourself and for the people around you [HY4]*”*.*

### 3.3 Theme 2: the trainer lets you practice everything that you want (QY0)

This theme reflects how mobility progress and engagement in training emerge from the interaction of collaborative goal-setting, individualized learning strategies, and skill practice embedded within engaging and appropriately challenging activities (what, personalisation, how challenging, and progression,). These key components of the training intervention are facilitated by trainers who establish positive relationships with PMWUs and possess the expertise required to maneuver a MWC and ensure safety (who provided).

#### 3.3.1 What and personalisation? training starts by choosing skills to achieve meaningful mobility goals

PMWUs appreciated setting their training goals, though some were unsure what they could achieve. Outcome measures completed prior to the intervention provided PMWUs with insight into what they could learn. Specifically, navigating the obstacles in the Wheelchair Skills Test helped certain participants, including some experienced PMWUs, identify “*which things I feel I can do, but cannot now [MY6]*”. The list of skills included in the WSTP and also part of this test was perceived as “*quite complete [QY0]*”, with PMWUs appreciating having a wide range of options, as illustrated by MY6 when describing what he liked most about the program: “*To have so many skills. If there were less, it would be less fun*”. Among the 30 skills, all were mentioned at least once either as skills PMWUs had chosen to practice or would have liked to practice, except “operates body positioning options” and “shifts weight”. The most often reported included rolling forward, going up and down inclines and curbs (e.g., sidewalks), getting over obstacles (e.g., door threshold), rolling on soft surfaces (e.g., grass), and wheelies. Managing the small slopes when rolling on sidewalks and negotiating gaps were additional skills, not part of the 30, that PMWUs valued practicing. Moreover, some PMWUs appreciated learning how to react in the case of a fall to prevent injuries: “*She taught me how to fall backwards. I know the technique so I don’t hit my head [QY5]*”. Other PMWUs perceived that watching videos of MWC users and discussing with the trainer which skills they could learn supported goal-setting: “*She showed me videos of the Youtube channel and asked me if I wanted to do the skill or not […] The Youtube channel can give many ideas of things to do. […] Videos should be shown earlier in the training process, as soon as the first session. [MY7]*”. Additionally, MC7 mentioned that rating the importance and performance of goals (i.e., Wheelchair Outcome Measure) further helped him goal-setting.

#### 3.3.2 What and personalisation? practice, demonstrations, and instructions broken down into small steps facilitate skill development

PMWUs emphasized that repeated skill practice was one of the elements that most fostered their mobility progress: “*That was really useful: repetition, always repetition. That's what helped me master it. [MC7]*”. When practicing skills, PMWUs liked using material (i.e., mirror, tapes on the floor, bubble wrap, cones) that provided them with feedback on how to position their wheelchair and on whether a skill was succeeded or not, as explained by MY6 when talking about descending high curbs in a wheelie position: “*The mirror, when I was going down so that I can see if my wheels align with the edge and the tape on the floor to check that I do not go too far […] They helped do it better, to get a sense whether I am well positioned or not*”. Performing skills in everyday life was considered important to retain or improve techniques, though some reported not practicing outside training sessions. Instructions on skills techniques, particularly when broken down into steps, were deemed helpful for PMWUs: “*For my transfer, I can do it. I follow my steps: look at my front wheels, place them correctly, put on my brakes, hold my hands, get down [QC9]*”. However, when reminded too frequently, instructions were described as annoying and infantilizing: “*Sometimes, she would tell me things that I already knew. I was thinking about it, but she told me and I did not like it because it made me feel like a baby. She would say it to me again and I applied it but she kept telling me, telling me [QC6]*”. PMWUs appreciated demonstrations, both in person and through videos, to understand how to position their wheelchair in space or their limbs on the wheelchair. Yet, in-person demonstrations were perceived as more reassuring when practicing skills PMWUs found scary, as illustrated by MY7 when describing what encouraged him to descend stairs: “*Having a demonstration in person not a video. I paid more attention to the things I was skeptical about, like the wheels and where they were positioned. That's what really scared me. I thought the big wheels would be off the ground*”*.*

#### 3.3.3 How challenging, progression, and personalisation? training is fun, particularly when it offers just-right challenges and is embedded into playful activities

All PMWUs perceived the WSTP as fun, enjoying skill practice and seeing their progress over time, as stated by QY8a-b when describing what made the training specific to the wheelie fun: “*I could not keep my balance 30 s on my rear wheels, but now it's fun I am able. I think it's fun to learn this skill [QY8a]. For me, it's the sandbox [rolling on soft surfaces: sand] that was fun. [QY8b]*”*.*

The level of challenge, related to skill difficulty, impacted PMWUs’ engagement. While some appreciated challenges or recognized that learning a skill could take time, others felt “*angry or sad maybe, cause it's hard skills [HC2]*”, “*bored doing hard things [QY3]*” or wanted to stop practicing “*Nooo! Noooo! [and stop skill execution in the middle of the incline] [QC2]*”. Providing achievable challenges that enabled them to push themselves, along with gradually increasing difficulty (e.g., practicing easier skills before harder ones), was described as important. To maintain PMWUs’ motivation during training, QY4 emphasized stopping skill practice at the right moment, as repeated failures could become discouraging.

Across all PMWUs, regardless of age or cognitive development, playful activities, including games, competitions, races, and obstacle courses, were consistently mentioned as making training enjoyable, whether integrated into skill practice or used as rewards. For example, a 9-year-old PMWU stated: “*You have to keep the games she made up to do the exercises [in the WSTP]. She hid animals. She said do this, do that, to find the animals. I liked it because it was like a hide-and-seek game. […] We had to practice our long pushes. She put animals on the ground and asked to do one push when reaching an animal. [QC6]*”. Such activities were perceived as even more engaging when they were based on interests or chosen by PMWUs. The use of sounds (e.g., bells, musical toys) as a reward was perceived as motivating by young PMWUs or PMWUs with cognitive limitations, as illustrated by a three-year-old who rolled forward to reach the trainer's car [target distance] to trigger the “*beep, beep, beep [QC2]*” sound of the car door locking. Music created a motivating training atmosphere: “*It was fun, especially when you put Eye of the Tiger on in the background. It motivated me and gave me a bit of adrenaline. [QY4]*”*.*

#### 3.3.4 Who provided? trainers have strong relational and communication skills, experience in navigating obstacles, and should collaborate with a MWC user as a peer trainer

The development of a strong connection with the trainer was consistently mentioned: “*The fact that she shared that with me told me that [trainer's name] had something wonderful… I don’t know how to explain it, but I felt it [QC9]*”*.* PMWUs described feeling comfortable during sessions, communicating easily and without shyness: “*I felt really comfortable and I even talked a lot [HY4]*”. Trainers were described as nice, smiley, funny, empathetic, encouraging, dynamic, and bringing a motivating energy, relational qualities PMWUs viewed as important when working with children and youth. PMWUs also indicated that clearly and patiently explaining skill techniques and being able to perform all MWC skills, even the most difficult ones, are essential trainer qualities: “*You have to be able to do pretty much everything, or at least know how to do it. You need to have some qualifications. Otherwise it would be boring: the person would never be able to explain what you’re supposed to do [MC7]*”. Recognizing that the capacity to navigate obstacles requires training and to support program continuity from research to clinical practice, some PMWUs suggested: “*Have more people trained to teach kids how to do it to be able to keep doing it for as long as possible with as many people as possible. I mean, it doesn’t just stop. The project needs to last more than one or two years [MY6]*”*.*

Although many considered their trainer to be the best person to deliver the WSTP, a few PMWUs indicated that anyone skilled in maneuvering a MWC, whether a rehabilitation professional or not, could teach them. Other PMWUs suggested that the ideal coaching team should consist of a rehabilitation professional and an experienced MWC user, with some expressing the interest to take on this role. The division of roles within this duo was explained by MY7: “*I can see myself as [trainer's name]'s assistant. I would do the skills the person wants to learn. If they want to go downstairs, go down an incline in a wheelie position, I would show them. [Trainer's name] would be behind me providing explanations. I could explain too, because what's interesting is that we have different visions. I would bring the day-to-day life aspects, the challenges we face, or when we’re asked what skills we would like to practice, I could give ideas*”. Trainers who are MWC users could be of any age but should have good communication skills and the desire to help others. Regarding safety, PMWUs specified that spotting has to be provided by “*a person on two feet [QY7]*”, because “*it's hard for someone in a wheelchair to follow others like a shadow [MC7]*”*.*

#### 3.3.5 Who provided? a good spotter only intervenes to ensure safety

Most PMWUs explained that having the trainer behind their wheelchair with a strap to support them if they lost control or fell (i.e., a spotter) was reassuring and enabled them to practice skills they would not have tried alone, as illustrated by QY1 when describing what made him feel confident during training: “*She told me that she was behind me, and she was holding me*”. They liked that the trainers let them try the skills by themselves, only intervening if needed for safety: “*Like with [trainer's name], you don’t really notice that she's there behind you. So that's why it's fun, because you really feel like you’re doing it without anyone behind you. Like, it doesn’t matter that she's there. She didn’t give me pushes or things like that. [MY6]*”. For certain advanced skills, such as ascending or descending stairs, PMWUs described feeling safer when two people were spotting them. Nonetheless, these safety measures were not sufficient to reassure a few PMWUs who reported fear and disliking practicing skills that involved removing anti-tippers or lifting the casters up: “*I did not like making me almost fall down [HY3]*”*.*

### 3.4 Theme 3: It depends on the person. Every child, every person with a disability or mobility aid is different. We're not all the same (HY6)

This theme reflects the importance for PMWUs to tailoring the how, where, when, and how much to their individual needs, emphasizing they all have different preferences. Regarding the who, perceptions on caregiver engagement in training varied. Harm emerged through discomfort during the practice of certain skills.

#### 3.4.1 How and personalisation? group training is motivating, while individual training enables personalisation

The individual format was appreciated by PMWUs for the intervention to be adapted to their needs, abilities, and characteristics: “*I think it's better to do it one-on-one, because it really makes it possible to follow the child. You can adapt more to the child. Even if you take two kids who have pretty similar abilities, which is rare, you know there are still so many variations in disability… [MC7]*”. Moreover, some PMWUs said being alone with the trainer helped them concentrate and feel more comfortable, as they are shy or would have been nervous about others watching them: “*no one else saw me miss it 20 times [QY4]*”. Others would have preferred dyad and group formats, describing them as “*more fun [HC7, HY4]*” and emphasizing opportunities to meet peers and support one another: “*We could give each other tips and motivate each other [QY1]*”. However, twins who received the WSTP together explained how being with someone else, especially siblings, could create a competitive atmosphere that sometimes interfered with their learning: “*The competitive spirit kicks in more when we’re both there. [QY8a]. That's true. There are things I am able to do and that QY8a can’t, and there are things he does and that I can’t. This is a competition. We try to do better than the other, but we are not able. We focus too much on the competition instead of training properly [QY8b]. Yes! [QY8a]*”*.*

#### 3.4.2 Where and personalisation? learning skill techniques in stable conditions, followed by practice in real-world settings

PMWUs valued learning skills in stable and controlled environments (i.e., the rehabilitation or research centre), followed by practice in real-world contexts (e.g., home, school, mall, library, parks, hiking trails): “*In the room at the rehabilitation center, there is no snow and other such things, so it's better to practice, but at the end we have to be able to do it in real conditions. […] For example, nine times at the rehabilitation center and three times at my place would be good. The rehabilitation centre is more for learning new skills. You learn three to four skills, and then after that you test them at home [MY6]*”. The inconveniences of the rehabilitation and research centers included the transportation burden on caregivers and increased time out of school, with PMWUs reporting that attending sessions added to their already numerous appointments: “*Towards the end, my dad was getting a bit tired of having to take us there because we were missing school. It was a lot for my parents, and the transportation costs as well [QY8a]*”. Variations in obstacle height, shape, and size were perceived as increasing difficulty in real-life situations, leading PMWUs to emphasize practicing the same skill across different obstacles, especially if training in only one setting: “*Even if you’re always training in the same place, try to find a spot with a different curb. In one place, you can still go ten meters farther, and there will be another curb not exactly the same size [QC4]*”*.*

#### 3.4.3 When and personalisation? training PMWUs soon after MWC provision, with follow-ups after wheelchair or environmental changes

According to some PMWUs who began using a wheelchair in early childhood and had the ability to reflect on how their mobility evolved over time, training should be offered soon after MWC provision, but not before pre-school years: “*I would say one year after receiving their wheelchair to get used to it, but not too young cause they would understand nothing. Minimum three years. It would have allowed me to learn basic things like backward, turn, rolling forward, things like that to be able to move around on my own more quickly, like at school or over short distances. I was able but not really good at it. [MY6]*”. Other moments PMWUs deemed critical for training included following changes in wheelchairs or environments: “*I want to do this training program again when I’m out of school and reaching post-secondary school because that's gonna come with a whole lot of new challenges that I'm gonna have to overcome and learn new skills. I think the wheelchair skills program would be a great asset to me, feeling more confident in the environment around me [HY6]*”. However, the weeks following an unplanned surgery were perceived as a less appropriate time for training by two PMWUs, with MC1 describing fatigue and pain when practicing skills that involved impacts with the ground (e.g., casters hitting the ground when losing balance during wheelie practice). While some PMWUs preferred training across different seasons to practice in varied conditions, others reported disliking or cancelling sessions due to seasonal weather-related challenges (e.g., snow, cold, rain, or heat waves).

PMWUs appreciated choosing session timing during the day and week to avoid conflicts with school and leisure activities and to train when they had more energy: “*Training was after school, but if possible, we tried to choose another day than the one I have swimming so that I was not too tired [QC6]*”. However, not everyone reported being provided with this opportunity, with certain moments reducing engagement in training: “*It was a little boring because I wanted to go to my school breaks. I wanted training to last 2 s to go back to my break [QY3]*”*.*

#### 3.4.4 How much and personalisation? individualized dosage rather than a fixed amount

The vast majority of PMWUs considered 12 sessions not enough, describing continued opportunities for learning and progress*:* “*Continue a few more sessions to get even better, because I feel like I was just starting to be good when the sessions ended. There would have been room for improvement if we would have continued for a few more weeks [QY7]*”. PMWUs emphasized the importance of flexibility, suggesting that the number of sessions be tailored to individual mobility levels (e.g., more sessions for novice users) or goal achievement to reach effective and confident wheelchair mobility for all: “*It depends on the person, but, I would say like five goals and once they master those five, getting a check-in every few years. The needs change over time [HY6]*”. Among PMWUs with the ability to understand time, preferred session duration ranged from 30 to 120 min, with most perceiving 45–60 min as sufficient to practice several skills without being overly physically demanding or boring. Moreover, session duration was recommended to be adapted to age, energy level, and the skills being practiced (e.g., stairs were reported as more demanding): “*Sometimes 45 min was enough and I was pretty fed up because I was tired, but other times it felt like we had just started, so I would’ve kept going for a few more minutes. It depended on how I felt that morning. Sometimes, I had more energy and sometimes I had less [QY7].*” Taking breaks as needed was perceived as key to managing energy and physical strain. Most PMWUs perceived that one to two sessions per week fit within families’ schedules while allowing time for rest and practice between sessions, with two sessions sometimes preferred to support skill development: “*Once a week, it was good, but when I had two close to another, it was easier because it was more fresh in my mind. […] Three, I would have missed too much school, I think. [QY1]*”*.*

#### 3.4.5 Who? caregivers can learn how to support mobility through observation, but their presence may be distracting

Different opinions about caregiver attendance (parents or tutors, siblings, grandparents) at the WSTP sessions were expressed by PMWUs, with some disliking, not minding or appreciating their presence. Some PMWUs mentioned that, through observing training sessions, caregivers saw what their children could do with their wheelchairs and learned how to support their day-to-day mobility, such as by providing encouragement, verbal cues, and reminders of the skill techniques: “*[Trainer's name] taught them things too. She taught them things to help me with my wheelchair. [QC9]*”. However, others reported being disrupted by caregivers, particularly by their reactions during skill practice: “*I preferred being alone. She was too nervous. I understand why, but I didn’t want her stress to rub off on me. You could tell just by the look on her face that she was nervous. When she was there, I had difficulty concentrating. I really really tried to focus on what I was doing, but I couldn’t [MY7]*”. With regards to supporting practice outside of training, PMWUs described how their parents could provide spotting, whereas some adolescents felt their parents may provide too much help or even do the skill for them instead of solely ensuring safety. An 11-year-old PMWU whose father attended two sessions explained they were planning to continue training together: “*We thought about my dad getting himself a MWC, so that we could train together. We are still looking for a MWC, but yeah we have a plan [QC4]*”*.*

#### 3.4.6 Harm: discomfort during the practice of certain skills

PMWUs did not report any negative impacts from training, although three children described discomfort when performing certain skills that did not persist after their execution. HC3 and HC5, who have cerebral palsy, reported “*pain*” and movements “*feeling not good*”, respectively, when casters hit the ground in ascending or descending curbs and due to fatigue in a spastic hand because of encouraged use. HC8, who has type 2 spinal muscular atrophy, expressed discomfort when performing skills that involved leaning the trunk forward, such as ascending inclines.

## 4 Discussion

The findings of this descriptive qualitative study, guided by the TIDieR-Rehab checklist (27), support the use of the WSTP for pediatric MWC skills training, suggesting benefits for self-directed mobility while aligning with PMWUs’ preferences. As such, this study contributes to the overarching goal of informing tailored WSTP interventions that enhance opportunities for PMWUs to receive evidence-based MWC skills training. To our knowledge, this is the first study to explore experiences with the WSTP and preferences for MWC skills training delivery from the perspectives of a maximum variation sample of PMWUs, giving a voice to a population rarely consulted regarding the rehabilitation interventions they receive (16, 17, 19). It offers a direct response to concerns previously raised by clinicians regarding the safety and feasibility of training community and advanced MWC skills in PMWUs (8, 35), as well as the appropriateness of the WSTP for pediatric populations (8).

Consistent with the results of our recent RCT (6) and with caregivers’ reported impacts of MWC skills training (12, 36), PMWUs described benefits from the WSTP. Perceived improvements in their skills and confidence in navigating obstacles with their MWC translated into greater autonomy and participation across home, neighborhood, daycare, school or work, and community settings. The WSTP also fostered safety when using the MWC by enhancing awareness of task demands and associated risks, as well as promoting greater understanding of capacities, with PMWUs recognizing which skill they mastered and which required further practice. These findings mitigate previous concerns reported by clinicians that PMWUs may become overconfident after training and attempt MWC skills beyond their capabilities (8). In addition, acknowledging that changes in their environments (e.g., entering a new school) may bring new mobility challenges, PMWUs raised the need for follow-up to sustain the impacts of WSTP training on their self-directed mobility and participation. Aligning with the “Future” component of the F-Words for Childhood Development framework (37), which informs many pediatric rehabilitation services, this study therefore suggests adopting a life-course approach to training to foster the acquisition, refinement, and consolidation of MWC skills over time.

Despite improved self-directed mobility following WSTP training, some PMWUs still received physical or unsolicited caregiver assistance despite being able to perform MWC skills independently. One possible explanation is that prolonged reliance on caregiver assistance may have conditioned PMWUs to seek help before attempting obstacles. Providing training soon after MWC provision, as recommended by PMWUs, may encourage greater initiative. Another explanation relates to the focus of the intervention on PMWUs alone, with caregivers observing rather than actively engaging in the training. Consistent with family-centred services (37), future interventions should address both PMWUs’ and caregivers’ needs, as well as family dynamics, to enhance self-directed mobility. The World Health Organization wheelchair provision guidelines (2023) recommend training both users and caregivers (1), and the WSTP includes considerations specific to caregivers that allow the use of a blended approach to training (10). Although this approach has not yet been evaluated in pediatrics, training PMWUs and caregivers together may promote a shared understanding of which skills can be performed independently and which require partial or complete assistance. For skills requiring partial assistance, such an approach could help PMWUs learn to direct assistance effectively while enabling caregivers to respond appropriately. For skills requiring complete assistance, caregivers could learn safe and efficient assistance techniques.

**Figure 1:**
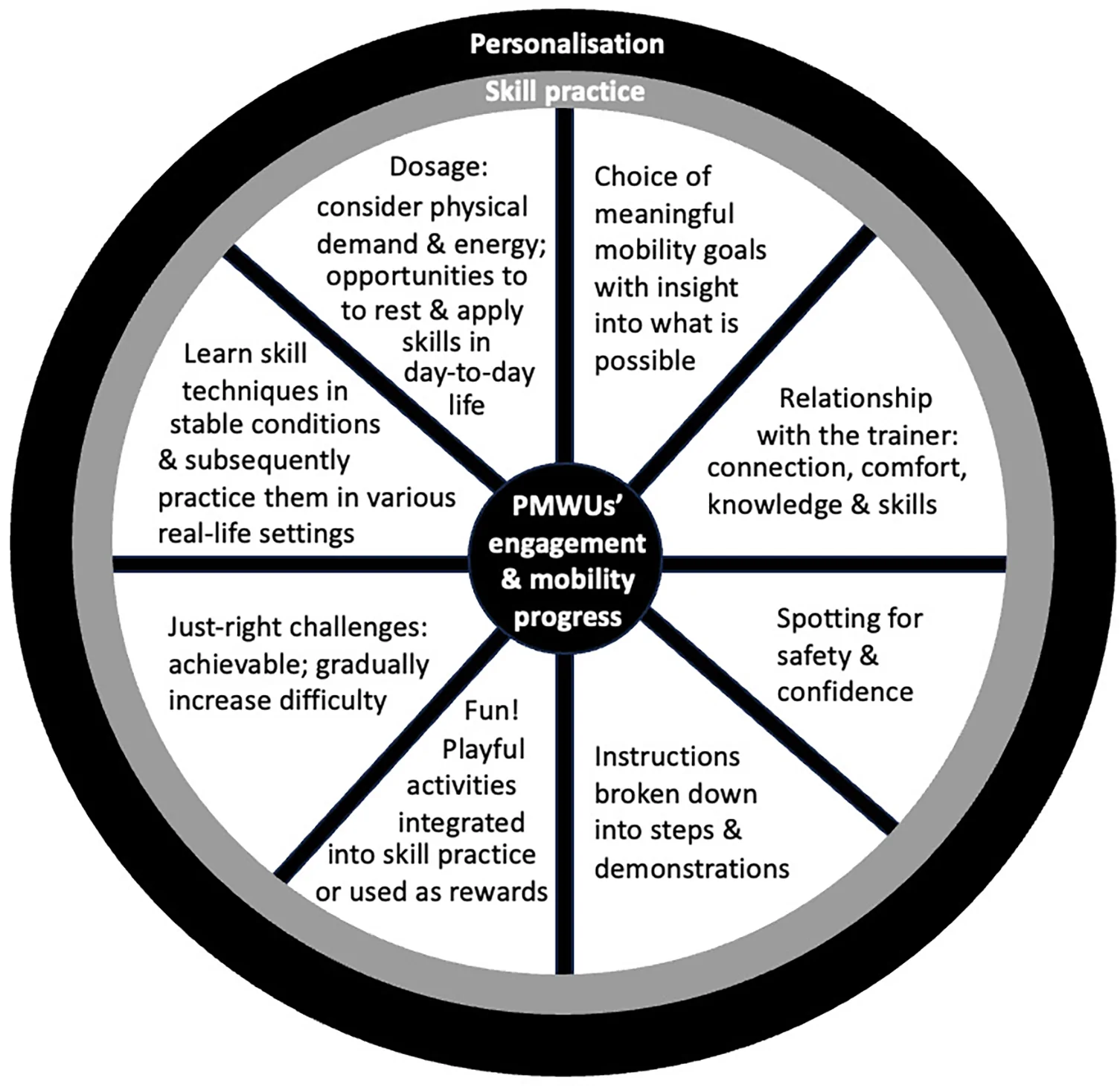
Ten most salient PMWU preferences for training delivery. The wheel in Figure 1 synthesizes PMWUs’ training preferences, including 10 key components to consider when delivering MWC skills training interventions in pediatrics. Personalisation and skill practice are illustrated as the tire and handrim, reflecting PMWUs’ perceptions of the importance of tailoring the intervention to individual needs and preferences and enabling repeated practice of skills to propel engagement and mobility progress.

Findings from the current study reveal that the WSTP aligns with PMWUs’ preferences for training delivery, further supporting its suitability. Given that training was tailored to their needs, PMWUs expressed positive experiences within the program to the extent that they would recommend it to peers and for it to become part of usual care. Figure 1 illustrates a wheel summarizing PMWUs’ most salient preferences for training delivery, which clinicians can take into account when planning training sessions. Personalisation and skill practice are depicted as the tire and handrim, as they represent the two key components driving engagement and mobility progress. Interestingly, all preferences are part of the WSTP, most of them within the WSTP 10 Trainer's Tools (10). For example, “learn skill techniques in stable conditions and subsequently practice in various real-life settings” is embedded within the variability of practice element of the Practice tool (10). This overlap between PMWU preferences and the WSTP further emphasizes that the program already incorporates key elements to deliver training in a manner that meets PMWUs’ needs and supports the development of their MWC skills. However, delivering this program appropriately for each PMWU, such as by selecting the most useful tools among the 10 to facilitate learning at different stages, requires sufficient training and ongoing support for trainers. For example, a combination of remote and hands-on training and monthly discussions with experts to answer questions and address challenges with training PMWUs, as was done in the RCT study (6, 29). The Pediatric Handbook, available online cost-free (38), and the pediatric-specific videos currently in development by members of our team may provide concrete guidance for applying the program in pediatrics.

Engagement in training and progress in self-directed mobility seemed to emerge from the interaction between meaningful goals, supportive trainer relationships, and personalised learning experiences rather than from skill practice alone. PMWUs described the strong relationships they built with trainers, who supported the achievement of meaningful mobility goals through instructions and demonstrations of skills techniques, repeated practice in both rehabilitation and real-world settings, and playful activities offering appropriate levels of challenge (i.e., just-right challenges). Studies on engagement in pediatric rehabilitation highlight the importance of connections to therapy goals, activities, and service providers, as well as the personalisation of interventions (16, 39, 40). To further enhance the relational and motivational aspects of training, some PMWUs suggested delivery of the WSTP through group and peer-led approaches. Older adults were satisfied with the WSTP intervention combining these two approaches, particularly because of the vicarious experience (i.e., if they can do it, I can too) and social aspects of the program (41). In fact, they reported feeling motivated and more confident by seeing the peer trainer and peer-learners achieve the skills (41). A feeling of belongness to the group through mutual understanding and the sharing of success and challenges was also reported (41). In pediatrics, single-group and case studies showed the positive outcomes of these approaches (5, 13, 42), but more rigorous studies are needed to demonstrate their effectiveness in improving self-directed mobility.

### 4.1 Reflections on the interview procedures conducted with PMWUs

The toolkit of customizable interview strategies were infrequently used to structure the interviews, as most PMWUs were comfortable responding directly to questions and motivated to share their experience to inform future program delivery. Vignettes and go-along interviews helped elicit aspects of experience that may not be spontaneously shared (e.g., elements disliked, suggestions for improvement), contributing to mitigate social desirability bias. However, drawing was infrequently chosen and may not have been the most empowering strategy due to fine motor difficulties perceived as limiting the illustration of PMWUs’ ideas. While caregiver-supported-interviews facilitated the contextualization of interview questions, some caregivers tended to prompt responses, potentially shaping what PMWUs expressed.

While the flexible interview procedures facilitated the exploration of PMWUs’ perceptions across diverse profiles, they did not fully support participants with limited speech abilities or those using augmentative and alternative communication. More adapted procedures could have been used, such as multiple interview encounters to build rapport and familiarize researchers with PMWUs’ modes of communication, sentence starters (e.g., “I like the trainer because she was…”), and photos to support recall or elicit contextual elements (43). Additionally, combining interviews with observational methods (e.g., video-recorded training sessions) could have helped understand these participants’ experiences and preferences (43).

### 4.2 Study limitations

Although interviewers emphasized that there were no right or wrong answers and invited suggestions to improve the WSTP, some PMWUs from the Halifax site, who were interviewed by their RCT trainer, may have been influenced by social desirability bias, particularly in response to questions about who delivered the intervention. While collaboration with trainers who knew the PMWUs best may have facilitated maximum variation sampling, it may also have influenced participant selection by excluding certain voices (e.g., PMWUs for whom training was challenging). Including PMWUs who did not complete the intervention may have mitigated this possibility. Regarding confirmability, team discussions helped ensure a shared understanding of participants’ perceptions (34). However, strategies such as summarizing interpretations of PMWUs’ experiences at the end of each interview and inviting participants to confirm, modify, or expand upon them, as done by Gagné-Trudel (2026), may have better supported grounding of the findings in participant data (44). Finally, the current findings reflect the perceptions of PMWUs from three settings across two Canadian provinces, and sociocultural and rehabilitation service differences may limit their broader transferability.

## 5 Conclusion

This descriptive qualitative study, based on the TIDieR-Rehab checklist (27), explored PMWUs’ experiences with the WSTP and their preferences for training delivery in pediatric rehabilitation settings, recognizing their right to be involved in discussions about their care. PMWUs reported positive experiences with the WSTP, and their preferences aligned closely with the program, supporting its use by clinicians to deliver tailored, evidence-based training. Findings suggest that effective pediatric MWC skills training depends not only on practicing MWC skills, but also on how training is structured and supported to create personalised, meaningful, and relationship-based learning experiences that empower PMWUs to develop the confidence and autonomy needed for self-directed mobility and participation in everyday life.

## Data Availability

De-identified raw data supporting the conclusions of this article will be made available by the authors upon request to the corresponding author.
