# Supplementary material for: Let us tell you how to train us! experiences of children and youth with the Wheelchair Skills Training Program and their preferences for training delivery: a descriptive qualitative study
Source: Front Rehabil Sci. 2026 Sep 3;7:1924405. doi: 10.3389/fresc.2026.1924405 (PMC13582389; doi:10.3389/fresc.2026.1924405)
Supplement: Supplementary file 1 [file Table1.docx]

Supplementary Material

# 1 Supplementary File 1. Semi-structured interview guide based on the TIDieR-Rehab checklist

| **TIDieR-Rehab items** | **Questions and probes** |
| --- | --- |
| **Why; What** | -What did you learn during training?   - Which activities/skills did you practice?   -Which activities/skills would you have liked to learn during training (things you wanted to do but did not do)?  -How did training help you use your manual wheelchair?   - What new things can you do with your wheelchair now?   -How did training improve your activities?   - How did training help you at (home, school, other activities they do)? - What can you do on your own now that you could not before training?   -Do you recommend the training program to other kids/teenagers? Why? |
| **Who; Personalisation** | -Who attended the training sessions with you? How did you feel when they were there?   - Would you have preferred them to leave or stay? Why?   -Who should come to the training sessions with you? Why? |
| **When** | -Your training sessions were (in the morning, afternoon, evening/week days, weekends). What do you think of this time/moment?   - If you could choose, at which moment in the day/week would you prefer training? Why?   -When should we offer training in kids’/teenagers’/youth’s life?   - What are the best moments for training? - You are [age] years old, would you have preferred to receive training when younger/older? - At which age? Why? |
| **What; Personalisation** | -How did you feel during your training?   - What was the most/least fun for you? - What can we do to make the training more fun for kids/teenagers? - What motivated/discouraged you to come to training? - Were there any moments you didn’t like during training? Why? - Did you ever feel (sad/angry/discouraged/scared) during training? When? What can we have done to make you feel better?   -What helped you learn the skills (things that helped you improve)? |
| **Who provided?** | -How was your trainer?   - What did you like/dislike about the trainer? - Did you feel comfortable with the trainer? What did the trainer do to make you feel comfortable/uncomfortable? - Which things would you change about the trainer (things they should change to be better at training skills)? - What are the qualities of a good trainer?   -Who are the best people to teach manual wheelchair skills?   - Other persons that could teach manual wheelchair skills - What do you think about being trained by (provide options if needed: occupational/physical therapists; caregivers; manual wheelchair users)? |
| **How?** | -In the sessions, you were alone with the trainer. How did you find that?   - What do you think about training with other kids/teenagers? |
| **Where?** | -At which places did you train (home, school, rehabilitation center)?   - What did you like/dislike about training there? - Which place was your favorite? Why? - Is there somewhere else you would have liked to train? Where? Why? |
| **How much?** | -What do you think of 45- to 60-min sessions?   - How long would you like the sessions to be? - Do you prefer longer/shorter sessions? Why? - What do you think of 30 mins, 1 hour and a half….?   -You received one/two sessions per week. How did you find that?   - If you could choose, how many times per week do you want to train? Why? - Would you like to train more/less often? Why? - What do you think of 1, 2, 3,… sessions/week?   -What do you think of 12 training sessions?   - You received [number] training sessions. If you could choose, how many would you have wanted? Why? - Do you want more/less sessions? Why? |
| **Harms** | -Do you have pain when you use your wheelchair?   - Where on your body? - Did you feel this pain before the training started?   -Did the training make this pain increase/decrease? |
| **General questions**  **(all items)** | -How did you find your manual wheelchair skills training?   - What did you like most in the training? - What should we keep/not change/is most important? - What did you like least in the training? - What should be improved?   -Would you like to train again? Why? |
| **General note:**  This is a semi-structured interview guide: questions should be asked depending on how the discussion unfolds, with some questions added, omitted, or rephrased as needed. | |
